# Biological Production of an Integrin *α*v*β*3 Targeting Imaging Probe and Functional Verification

**DOI:** 10.1155/2015/681012

**Published:** 2015-01-15

**Authors:** Mi-Hye Hwang, Jung Eun Kim, Sang-Yeob Kim, Senthilkumar Kalimuthu, Shin Young Jeong, Sang-Woo Lee, Jaetae Lee, Byeong-Cheol Ahn

**Affiliations:** ^1^Department of Nuclear Medicine, Kyungpook National University School of Medicine, 50 Samduk-dong 2-ga, Jung Gu, Daegu 700-721, Republic of Korea; ^2^Department of Biochemistry and Cell Biology, Kyungpook National University School of Medicine, Daegu 700-842, Republic of Korea

## Abstract

The aim of the present study is to establish a bacterial clone capable of secreting an integrin *α*v*β*3 targeting probe with bioluminescent and fluorescent activities, and to verify its specific targeting and optical activities using molecular imaging. A bacterial vector expressing a fusion of secretory Gaussia luciferase (sGluc), mCherry, and RGD (sGluc-mCherry-RGDX3; GCR), and a control vector expressing a fusion of secretory *Gaussia luciferase* and mCherry (sGluc-mCherry; GC) were constructed. The GCR and GC proteins were expressed in *E. coli* and secreted into the growth medium, which showed an approximately 10-fold higher luciferase activity than the bacterial lysate. Successful purification of GCR and GC was achieved using the 6X His-tag method. The GCR protein bound with higher affinity to U87MG cells than CHO cells in confocal microscopy and IVIS imaging, and also showed a high affinity for integrin *α*v*β*3 expressing tumor xenografts in an *in vivo* animal model. An *E. coli* clone was established to secrete an integrin *α*v*β*3 targeting imaging probe with bioluminescent and fluorescent activities. The probe was produced feasibly and at low cost, and has shown to be useful for the assessment of angiogenesis *in vitro* and *in vivo*.

## 1. Introduction

The formation of new blood vessels in a process termed angiogenesis is essential for the growth and metastasis of a tumor. Early in tumorigenesis, an “angiogenic switch” triggered by hypoxia results in the activation of oncogenes and/or metabolic stress [1]. Integrin *α*v*β*3, which plays a pivotal role in angiogenesis, is overexpressed in activated endothelial cells during angiogenesis, whereas it is absent in quiescent endothelial cells and normal tissues [2]. The restricted expression of integrin *α*v*β*3 in tumors makes it a suitable receptor for tumor targeting. Antiangiogenic agents reduce blood flow and vascular permeability and are therefore important in the treatment of cancer [3]. Many preclinical studies and clinical trials have demonstrated that inhibition of angiogenesis is an effective method to control tumor growth, supporting the development of antiangiogenesis therapeutics [4].

The therapeutic use of the integrin *α*v*β*3 targeting motif RGD (Arg-Gly-Asp) is an effective method for regressing tumor masses without harmful effects to normal tissues [5]. In the past decade, the efficacy of several RGD-containing molecular imaging tracers monitored and tumor integrin *α*v*β*3 expression has been evaluated using single photon emission computed tomography, positron emission tomography, magnetic resonance imaging (MR), optical imaging, and ultrasonography imaging [6]. Noninvasive determination of integrin *α*v*β*3 expression can be used to evaluate the efficiency of antiangiogenic drug candidates [7].

Integrin *α*v*β*3 targeting molecular imaging tracers consist of two functional domains, a targeting moiety with affinity to integrin *α*v*β*3 and a signaling component such as a radioisotope, fluorophore, or microbubble. The use of this molecular imaging strategy to investigate the process of angiogenesis or to assess the effect of antiangiogenic drugs requires the preparation of tracers at every time point of the experiment, which involves laborious and extensive experimental steps. Thus, there is a pressing need for feasible methods for the production of angiogenesis-targeting probes.

Gaussia luciferase (Gluc, 185 aa, and 19.9 kDa) was derived from the marine copepod* Gaussia princeps*, which emits light at a peak of 480 nm when interacting with the appropriate substrates [8]. This secreted protein produces a bioluminescent signal inside and outside cells without requiring ATP [9]. This study hypothesized that a fusion protein consisting of Gluc and RGD may be effective for targeting and visualizing the expression of integrin *α*v*β*3. In the present study, we constructed a bacterial plasmid vector expressing an integrin *α*v*β*3 targeting peptide probe with optical reporter activity and verified its potential as an optical probe specifically targeting integrin *α*v*β*3.

## 2. Materials and Methods

### 2.1. Construction of the Expression Vector for Fusion Protein in* E*.* coli* Cells

The secretory* Gaussia luciferase* gene (Gaussia luciferase with a secretory sequence; sGLuc) was kindly provided by professor Jung-Jun Min (Chonnam National University, Republic of Korea). The sGluc cDNA was amplified from plasmid pcDNA3.1-sGluc using the forward primer 5′-CGGGATCCTATGGGAGTCAAAGTTCTGTTT and reverse primer 5′-CGGAATTCTTACTCGAGACCAGATCTACCGGTACCGTCACCACCGGCCCCCTTGATCT-3′. Amplification products were subcloned downstream of the internal T7 promoter between the EcoR I and BamH I sites of the pRSET vector (Invitrogen, Carlsbad, CA, USA) to produce pRSET-sGluc. The mCherry fluorophore attached to three copies of Arg-Gly-Asp (RGDX3) was subcloned behind the T7 promoter between the KpnI and XhoI sites of pRSET-sGLuc to produce pRSET-sGluc-mCherry-RGDX3 (GCR). A control vector expressing a fusion protein of sGluc and mCherry without the RGD motif (sGluc-mCherry: GC) was also constructed. Isolated plasmids were analyzed for the presence of inserts, and positive clones were confirmed by the presence of the insert based on fragment size using restriction enzyme analysis and by DNA sequencing.

### 2.2. Expression and Purification of the GCR and GC Fusion Proteins from* E. coli* Cells

The GCR or GC expression vector was transformed into competent JM109* E. coli* cells and synthesis of the GCR or GC fusion protein was induced with isopropyl-*β*-thiogalactopyranoside (IPTG). The seed culture of* E. coli* cells containing the expression vectors pRSET-GCR and pRSET-GC was grown in Luria-Bertani (LB) broth containing ampicillin (50 *μ*g/mL) at 37°C. The expression of the 6X His-tagged GCR and GC proteins was induced by 1 mM IPTG for 4 h at 37°C and the tagged proteins were purified with a Ni-NTA resin (Qiagen, Valencia, CA, USA) according to the manufacturer's recommendation. Protein concentration was determined by the bicinchoninic acid (BCA) method using a commercially available kit and bovine serum albumin as a standard (Pierce, Rockford, IL, USA). The purified proteins were analyzed by 12% sodium dodecylsulfate-polyacrylamide gel electrophoresis (SDS-PAGE). The purified GCR and GC fusion proteins were analyzed by bioluminescent imaging with IVIS Lumina II (Caliper Life Sciences, Hopkinton, MA, USA) after treatment with coelenterazine (1 *μ*g in 100 *μ*L of Saline) (Promega, Madison, WI, USA). The area of the region of interest (ROI) was kept constant and luminescence signals were expressed as photons/sec/cm^2^/sr.

### 2.3. Cell Lines and Culture Conditions

Human glioblastoma-astrocytoma epithelial-like (U87MG) cells expressing endogenous integrin *α*v*β*3 and Chinese hamster ovary cells (CHO) without integrin *α*v*β*3 expression were grown in RPMI-1640 media (Hyclone, Logan, UT, USA) containing 10% fetal bovine serum (Hyclone, Logan, UT, USA) and 1% penicillin-streptomycin and cultured at 37°C in a 5% CO_2_ atmosphere. To establish stable cell lines with enhanced green fluorescent protein (EGFP) expression (CHO-EGFP and U87MG-EGFP), U87MG cells and CHO cells were grown to 70−80% confluence and transfected with an EGFP expression vector (pEGFP-N1, Clontech, Mountain View, CA, USA) using Lipofectamine 2000 (Invitrogen, Carlsbad, CA, USA) according to the manufacturer's guidelines. The cells were further treated with 700 *μ*g G418 (GIBCO, Carlsbad, CA, USA) for 2 weeks to select cells with EGFP expression.

### 2.4. Determination of Luminescence Activity

Bacterial cells (*E. coli*; JM109) transformed with the plasmid pRSET-GCR and pRSET-GC were grown overnight at 37°C in LB medium. The luciferase activity of the medium and the cells were measured separately. The luciferase activity of cells was determined in cell lysates using a microplate luminometer (Molecular Devices, Sunnyvale, CA, USA) with the Renilla luciferase reporter assay system (Promega, Madison, WI, USA) according to the manufacturer's instructions. The luciferase activity was expressed as relative luminescence units (RLU) and normalized to the protein concentration determined by the BCA method.

### 2.5. *In Vitro* Integrin *α*v*β*3 Binding Study: Confocal Imaging

U87MG-EGFP and CHO-EGFP cells were grown in collagen-coated 35 mm glass-base dishes (Asahi techno glass, Tokyo, Japan) for 24 h in growth medium. Subsequently, the cells were washed with PBS and fixed with 4% paraformaldehyde at 4°C for 30 min. The cells were then incubated with the purified GCR or GC fusion proteins (2 *μ*g) at 37°C for 1 h and washed two times with PBS. Confocal scanning laser microscopy was performed with a Zeiss LSM 510 instrument (Carl Zeiss, Oberkochen, Germany) with a 40x objective. The images were processed using Aim Image Examiner software (Carl Zeiss, Oberkochen, Germany).

### 2.6. *In Vitro* Integrin *α*v*β*3 Binding Study: Bioluminescent Imaging

One day before bioluminescent imaging, CHO and U87MG cells were seeded in 24-well plate (1 × 10^5^ cells per well) and then incubated with 2 *μ*g of the purified protein (GCR or GC) for 1 h at 37°C. After washing with PBS, coelenterazine (1 *μ*g in 200 *μ*L of PBS) (Promega, Madison, WI, USA) was added into the wells and the plate was imaged with IVIS Lumina II. An ROI was drawn manually over each well. The size of the ROI was kept constant and a bioluminescent signal was obtained and expressed as photons/sec/cm^2^/sr.

### 2.7. *In Vivo* Bioluminescence Imaging

All animal experimental protocols were approved by the Committee for the Handling and Use of Animals of Kyungpook National University. An* in vivo* imaging study was performed with 20 mice and separated into four groups. Each group containing 5 mice used the following procedures: U87MG and CHO cells (5 × 10^6^ cells/100 *μ*L) were subcutaneously injected in the right thigh of BALB/c nude female mice (*n* = 5; Japan SLC Inc.). After 14 days, GCR or GC protein (20 *μ*g in 100 *μ*L of saline) was injected into tumor-bearing mice via the tail vein and 1 h after the injection coelenterazine (20 *μ*g in 100 *μ*L of saline) was injected, and subsequently bioluminescent imaging (Promega, Madison, WI, USA) was done by using IVIS Lumina II. Regions of interest were drawn manually over xenograft tumors, and bioluminescent signal intensity was measured and expressed as photons/sec/cm^2^/sr.

### 2.8. Statistical Analysis

All numerical data were expressed as the mean ± standard deviation. Intergroup differences were assessed using a two-tailed Student's *t*-test. *P* values <0.05 were considered statistically significant.

## 3. Results

### 3.1. Construction of the Expression Vector for Fusion Proteins

**Figure 1:**
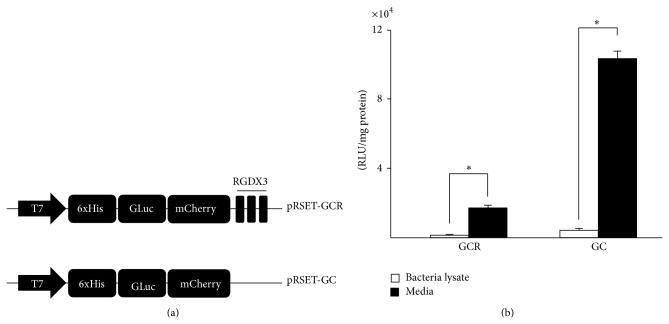
Vector constructs for GCR and GC proteins and functional assay of the GCR and GC proteins. (a) Schematic representation of plasmid vectors expressing GCR or GC proteins. The vector was designed to produce a fusion protein consisting of Gluc, mCherry, and the RGD motif, or Gluc and mCherry driven by the T7 promoter. (b) The luciferase activity of the medium and the cell lysates was measured for verification of successul production of the fusion proteins by the expression vectors. The luciferase activity of the growth medium was higher than that of the bacterial lysate (^*^ *P* < 0.05). GCR, sGluc-mCherry-RGDX3; GC, sGluc-mCherry; sGluc, secretory Gaussia luciferase; RGD, Arg-Gly-Asp.

A bacterial expression vector with the fusion genes of Gluc, mCherry, RGD motif (pRSET-GCR) and Gluc, mCherry, without RGD motif (pRSET-GC), was constructed (Figure 1(a)) and transformed into* E. coli* JM109 to produce the GCR and GC proteins, which were secreted into the growth medium. The luciferase activity of the growth medium containing the GCR and GC proteins was approximately 10- and 16-fold higher, respectively, than that of the bacterial lysate (Figure 1(b)).

### 3.2. Purification of GCR and GC Fusion Proteins from* E. coli* Cells

**Figure 2:**
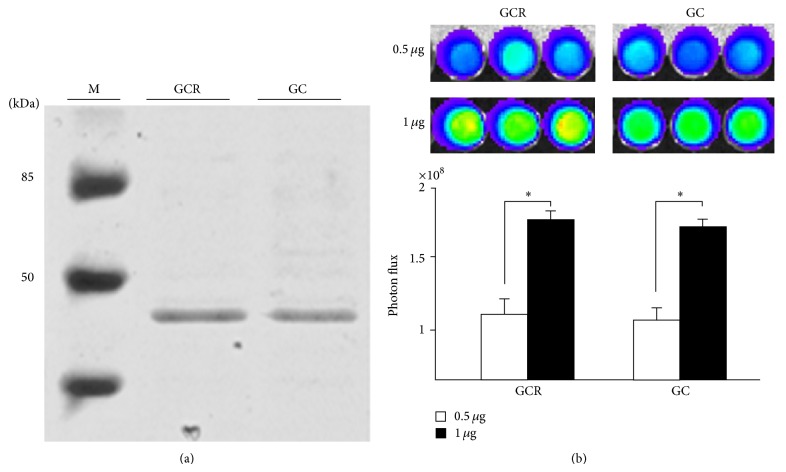
Image showing the SDS-PAGE separation of the purified GCR and GC proteins. (a) The molecular weight ladder is shown in the left lane. The sizes of the GCR and GC proteins were determined as 48 kDa. (b) Bioluminescence imaging of purified GCR and GC fusion proteins. Luciferase activity was positively correlated with protein concentration (0.5 and 1 *μ*g) (^*^ *P* < 0.05). SDS-PAGE, sodium dodecylsulfate-polyacrylamide gel electrophoresis.

GCR and GC protein expression was induced with IPTG and the 6X His-tagged proteins were purified by immobilized metal affinity chromatography on Ni-NTA columns. The purified products were analyzed by SDS-PAGE (Figure 2(a)), which detected bands of 48 kDa for the GCR and GC proteins. The luciferase activities of the purified proteins increased with protein concentration (Figure 2(b)).

### 3.3. *In Vitro* Binding of GCR Protein to Integrin *α*v*β*3 Positive Cells

**Figure 3:**
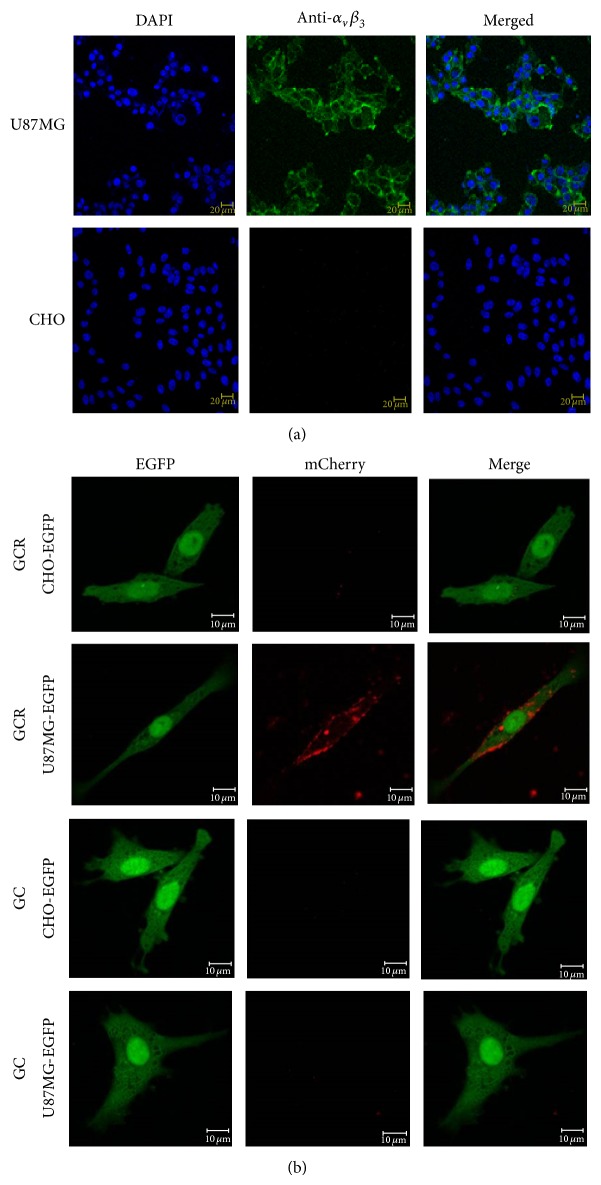
(a) Endogenous integrin expression using immunostaining in CHO and U87MG cells. (b) Confocal fluorescence microscopy images of cells with or without integrin *α*v*β*3. U87MG-EGFP and CHO-EGFP cells were incubated with 2 *μ*g of GCR or GC protein at 37°C for 1 h. Confocal microscopy imaging following treatment with the GCR protein revealed fluorescent signals from U87MG-EGFP cells but no signals from CHO-EGFP cells. Confocal microscopy following treatment with the GC protein revealed no fluorescent signals from U87MG-EGFP or CHO-EGFP cells. Scale bar corresponds to 10 *μ*m. U87MG, human glioblastoma-astrocytoma, epithelial-like cells; CHO, Chinese hamster ovary cells; EGFP, enhanced green fluorescent protein.

The* in vitro* binding affinity of the fusion proteins to the integrin *α*v*β*3 receptor was tested on integrin *α*v*β*3 positive U87MG cells and negative CHO cells. In the presence of the GCR protein, the mCherry signal was detected on the cell membrane of integrin *α*v*β*3 expressing U87MG-EGFP cells whereas no or negligible signal was detected in CHO-EGFP cells under the same conditions (Figure 3(a)). U87MG-EGFP and CHO-EGFP cells incubated with the GC protein did not show a detectable fluorescent signal (Figure 3(b)).

**Figure 4:**
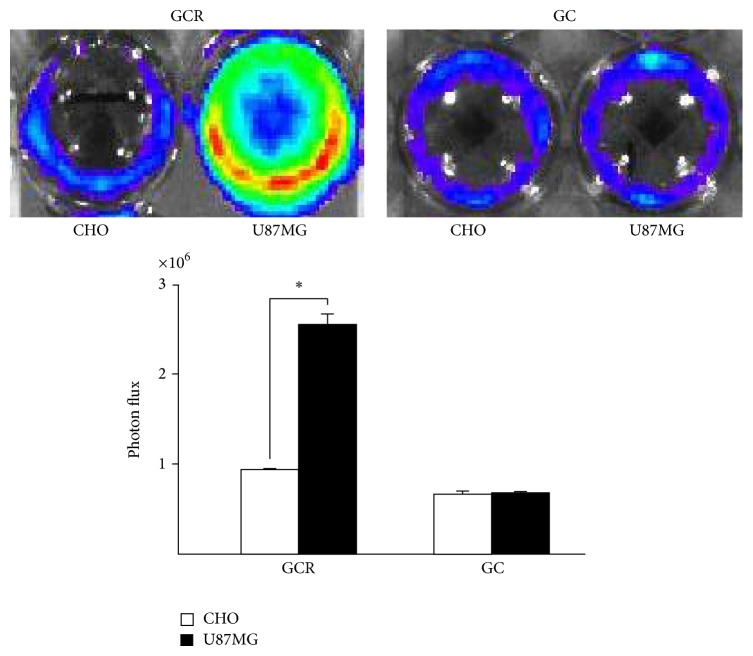
After incubation with the GCR protein, U87MG cells showed 3-fold higher luciferase activity than CHO cells (^*^ *P* < 0.05). Negligible signals were detected for both U87MG and CHO cells after incubation with the GC protein.

Luciferase activity was 3-fold higher in U87MG cells than in CHO cells after incubation with the GCR protein for 1 h, and negligible signals were detected in both CHO and U87MG cells after 1 h incubation with the GC protein (Figure 4).

### 3.4. *In Vivo* Bioluminescent Imaging

**Figure 5:**
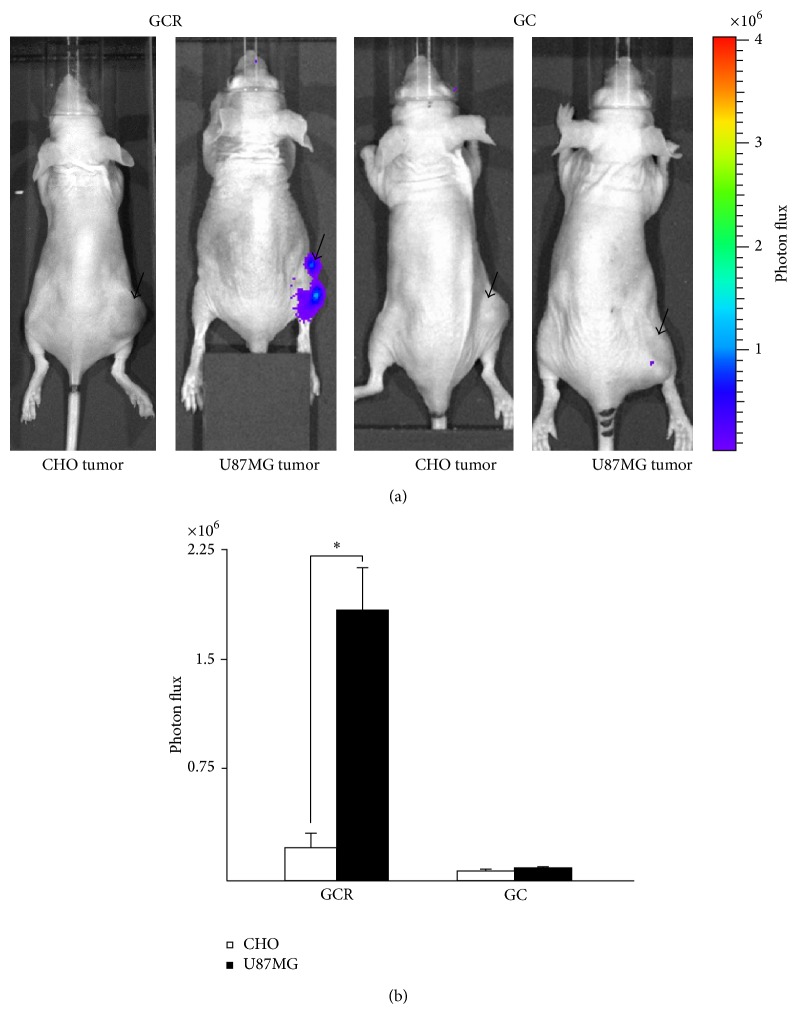
(a)* In vivo* bioluminescent imaging of xenografts with or without integrin *α*v*β*3. U87MG tumors expressing integrin *α*v*β*3 were visualized after systemic injection of GCR protein and coelenterazine, whereas CHO cell xenografts without integrin *α*v*β*3 were not detectable. Both tumor xenografts showed no signals after injection of GC protein and coelenterazine. (b) The total photon counts at the ROIs of U87MG and CHO tumor xenografts were measured and compared (^*^ *P* < 0.05). Black arrow shows tumor site. ROI, region of interest.

*In vivo* imaging experiments were performed by intravenous injection of the GCR and GC proteins into U87MG and CHO tumor-bearing mice. The tumor targeting specificity of the GCR protein was confirmed by bioluminescent imaging after injection of coelenterazine. Similar to our* in vitro* results showed that the high affinity of GCR for integrin *α*v*β*3-positive tumor cells, systemically administered GCR protein produced a considerably stronger bioluminescent signal in integrin *α*v*β*3-positive U87MG tumors than in integrin *α*v*β*3-negative CHO tumors (Figure 5(a)). The total photon counts in GCR treated U87MG and CHO tumors were 1.7 ×10^6^ ±2.7 ×10^5^ and 2.87 ×10^5^ ± 8.97 ×10^4^ photons/sec/cm^2^/sr, respectively, and those in GC treated U87MG and CHO tumors were 7.8 ×10^4^ ± 4.8 ×10^4^ and 6.0 ×10^4^ ± 2.5 ×10^4^ photons/sec/cm^2^/sr, respectively (Figure 5(b)).

## 4. Discussion

Angiogenesis is a complex process consisting of the formation of new capillary blood vessels, and it is regulated by pro- and antiangiogenic factors [10]. Tumors require a constant supply of oxygen and nutrients to support growth and angiogenesis is therefore one of the hallmarks of cancer [11]. Inhibition of angiogenesis is a promising strategy for the treatment of cancer and its clinical application has been investigated extensively [12]. Several molecular angiogenic markers that are overexpressed in tumors, such as integrin, particularly *α*v*β*3, endoglin, and vascular endothelial growth factor receptor 2 are potential targets for early cancer detection [13–15]. Integrin *α*v*β*3 is highly expressed in activated tumor endothelial cells and plays critical roles in the proliferation and movement of vascular endothelial cells, angiogenesis, and tumor growth [16]. The RGD peptide recognizes the integrin *α*v*β*3 receptor and is an effective and specific targeting probe for imaging tumor growth and survival, angiogenesis, and metastasis [17]. Over the past decade, most studies which aimed at identifying candidate therapeutic agents relied on* in vitro* angiogenesis assays that assess the effects of drugs on endothelial cell migration, proliferation, apoptosis, and tube formation [18]. Targeting probes can be used to screen antiangiogenic drugs and determine the efficacy of selected antiangiogenic drugs in comparison with conventional chemotherapy* in vivo* [19].

Recently, several investigators have reported the use of various RGD ligand-containing molecular probes for MR, nuclear imaging, and optical imaging. The use of MR imaging for monitoring therapeutic responses in integrin *α*v*β*3 positive tumor-bearing mice has been reported [20]. Zhang et al. recently performed MR imaging of integrin *α*v*β*3 using RGD-conjugated magnetic particles termed USPIOs [21]. An ^18^F-galacto-RGD probe was developed for positron emission tomography imaging of angiogenesis with high sensitivity [22]. Despite their value, the use of RGD-conjugated nanoparticles or radioisotopes is limited by the laborious and expensive processes involved in their production and quality control. In an effort to solve these problems, we have developed a feasible, cost effective, and convenient system for the production of integrin *α*v*β*3 targeting optical probes.

Yan et al. fused three copies of the RGD sequence to truncated tissue factor (tTF) to produce the fusion protein (RGD)3/tTF for specific binding to integrin *α*v*β*3* in vitro* and they demonstrated that the addition of RGD binding motifs to tTF increases the binding ability of RGD/tTF to integrin *α*v*β*3* in vitro*, thus improving the targeting of angiogenic sites [23]. They speculated that the fusion protein might localize to target sites more effectively and rapidly in an* in vivo* environment; however, they did not provide* in vivo* data. Imaging signal producing proteins with the RGD motif can be developed as effective probes for the delivery of proteins to angiogenic sites in* in vivo* studies. Therefore, we fused the optical reporter protein Gluc to RGD peptides to generate an imaging probe and visualized the* in vivo* distribution and specific delivery of the fusion protein to angiogenic sites without animal sacrifice.

In the present study, we constructed a plasmid vector expressing a noble fusion protein composed of three copies of the RGD sequence, and a fluorescent and luminescent protein was successfully produced in genetically engineered bacteria. The benefits of Gluc include its enhanced brightness and stability over other luciferases. In addition, Gluc uses substrate coelenterazine whereas firefly luciferase (Fluc) uses D-luciferin, allowing simultaneous monitoring of tumors labeled with Fluc [24]. The fluorescent protein mCherry was also included in the probe to achieve cellular level resolution of images. Our results indicated that Gluc and mCherry may be used for signal detection in* in vitro* microscopic and* in vivo* macroscopic experiments. We verified the integrin *α*v*β*3 targeting efficacy of the probe by using* in vitro* molecular imaging techniques such as the luciferase assay, confocal microscopy, and bioluminescence imaging. We obtained high resolution confocal microscopy images to examine integrin *α*v*β*3-RGD clusters in the cell membrane, which was further confirmed by bioluminescence imaging. As shown in Figure 4, bioluminescence imaging clearly demonstrated that the RGD motif fusion protein showed 3-fold higher binding specificity to integrin *α*v*β*3 positive (U87MG) than negative cells. The activity and binding properties of the fusion protein probe were evaluated* in vivo* in a mouse xenograft model, which showed that the total photon counts after treatment with the GCR protein were significantly higher in integrin *α*v*β*3 expressing U87MG tumors than in integrin *α*v*β*3-negative CHO tumors (Figure 5(b)). This result can be attributed to the fact that GCR can preferentially interact with integrin *α*v*β*3-positive U87MG tumors. Therefore, the new fusion protein probe should have useful applications, similar to conventionally produced RGD imaging probes for targeted diagnosis and therapy of integrin *α*v*β*3-positive tumors.

In the present study, we describe a feasible bacterial production system for integrin *α*v*β*3 targeting probes, which has the following advantages: first, a low production cost compared with labeling of nuclear, MR, or fluorophore signaling component to RGD and second, the availability and easy collection of the probe from the bacterial medium. The bacterial system described in this study for the production of noble optical imaging probes will enable the evaluation of angiogenesis* in vitro* and* in vivo*.

## 5. Conclusion

We established an* E. coli* clone that secretes an integrin *α*v*β*3 targeting imaging probe containing bioluminescent and fluorescent activities. The probe was produced at low cost and feasibly and could be useful for evaluating angiogenesis in* in vitro* and* in vivo* studies.
